# SPATA4 improves aging‐induced metabolic dysfunction through promotion of preadipocyte differentiation and adipose tissue expansion

**DOI:** 10.1111/acel.13282

**Published:** 2020-12-13

**Authors:** Zhongchi Li, Kang Xu, Sen Zhao, Yannan Guo, Huiling Chen, Jianquan Ni, Qingfei Liu, Zhao Wang

**Affiliations:** ^1^ Protein Science Key Laboratory of the Ministry of Education School of Pharmaceutical Sciences Tsinghua University Beijing China; ^2^ Key Laboratory of Big Data for Spinal Deformities Peking Union Medical College Hospital Beijing China; ^3^ School of Medicine Tsinghua University Beijing China

**Keywords:** adipocyte differentiation, aging, fat redistribution, SPATA4

## Abstract

Spermatogenesis‐associated protein 4 (SPATA4) is conserved across multiple species. However, the function of this gene remains largely unknown. In this study, we generated *Spata4* transgenic mice to explore tissue‐specific function of SPATA4. *Spata4* overexpression mice displayed increased subcutaneous fat tissue compared with wild‐type littermates at an old age, while this difference was not observed in younger mice. Aging‐induced ectopic fat distribution, inflammation, and insulin resistance were also significantly attenuated by SPATA4. In vitro, SPATA4 promoted preadipocyte differentiation through activation of the ERK1/2 and C/EBPβ pathway and increased the expression of adipokines. These data suggest SPATA4 can regulate lipid accumulation in a tissue‐specific manner and improve aging‐induced dysmetabolic syndromes. Clarifying the mechanism of SPATA4 functioning in lipid metabolism might provide novel therapeutic targets for disease interventions.

## Graphical abstract

SPATA4 promotes preadipocyte differentiation through activation of ERK1/2 and C/EBPβ pathway, attenuates aging‐induced ectopic distribution of fat, and improves metabolic homeostasis.

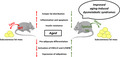

## 1 INTRODUCTION

Fat redistribution, wherein fat tissue transfers from subcutaneous depots to visceral depots, such as liver, muscle, and heart, occurs commonly in aging individuals. This phenomenon is associated with increased risk of the dysmetabolic syndromes, such as diabetes, hypertension, and hyperlipidemia, as well as various other aging‐related diseases (Palmer & Kirkland, 2016; Pararasa et al., 2015). Preadipocyte replication and differentiation, susceptibility to apoptosis, and inflammatory cell infiltration vary among depots (Tchkonia et al., 2010). Subcutaneous preadipocytes possess greater replicative potential than omental preadipocytes and aging‐induced decrease of replicative ability only appears on subcutaneous adipocytes. Additionally, abdominal subcutaneous preadipocytes have a greater capacity for adipogenesis along with higher expression of PPARγ and C/EBPα, compared with omental preadipocytes (Tchkonia et al., 2005; Van Harmelen et al., 2004). Because obesity and aging are associated with sustained activation of fat tissue immune response, increased lipolysis, and decreased intake of fatty acid (Palmer & Kirkland, 2016; Pararasa et al., 2015), the maintenance of the appropriate distribution and function of fat tissue represents a promising strategy for the management of dysmetabolic syndromes.

Spermatogenesis‐related gene 2 (SRG2) was first cloned from a cryptorchidism mouse model. The human homolog of SRG2, spermatogenesis‐associated protein 4 (SPATA4, previously named TSARG2), was identified in human testes at different stages and was significantly upregulated in cryptorchidism (Liu, Lu, et al., 2004). Following research revealed that it was expressed in and highly conserved across multiple species (Liu et al., 2004, 2005; Xie et al., 2007). We have previously reported that SPATA4 contains a calponin homology (CH) domain at the N‐terminus, and is negatively modulated by regulatory factor X1 (RFX1) (Jiang et al., 2013). In vitro, SPATA4 promotes osteoblast differentiation through the ERK‐activated RunX2 pathway (Wang et al., 2011) and protects Hela cells from etoposide‐induced apoptosis through the mitochondrial apoptotic pathway (Jiang et al., 2015). Together, these studies suggest that SPATA4 plays a role in cell differentiation.

Several studies have showed that the secretion of adipokines from adipose tissue regulates reproductive function (Landry et al., 2013) and that obesity has a negative impact on male reproduction (Teerds et al., 2011). Importantly, SEIPIN, a transmembrane protein localizing at the endoplasmic reticulum, causes both dysregulation of adipose tissue as well as male sterility (Jiang et al., 2014). *Spata4* is highly conserved in the reproductive organs of various species, but the function of SPATA4 on metabolism remains unknown. Here, we overexpressed *Spata4* ubiquitously in mice to explore its role in different tissues and organs. SPATA4 overexpressing mice maintained subcutaneous fat mass and insulin sensitivity in old age compared with wild‐type mice. In vitro, SPATA4 stimulated the activation of ERK1/2 and C/EBPβ, promoted differentiation of preadipocytes, and increased expression of adipokines. Unexpectedly, we did not observe any significant phenotypical changes in reproductivity, appearance, and metabolism in *Spata4* knock out mice. These results unveil a role of SPATA4 in adipocyte differentiation and fat tissue distribution. Clarification of the mechanisms by which SPATA4 regulates fat metabolism would be beneficial to our understanding of how aging and obesity affect the development of dysmetabolic syndrome.

## 2 RESULTS

### 2.1 *Spata4* overexpression attenuates aging‐induced body weight loss

**FIGURE 1:**
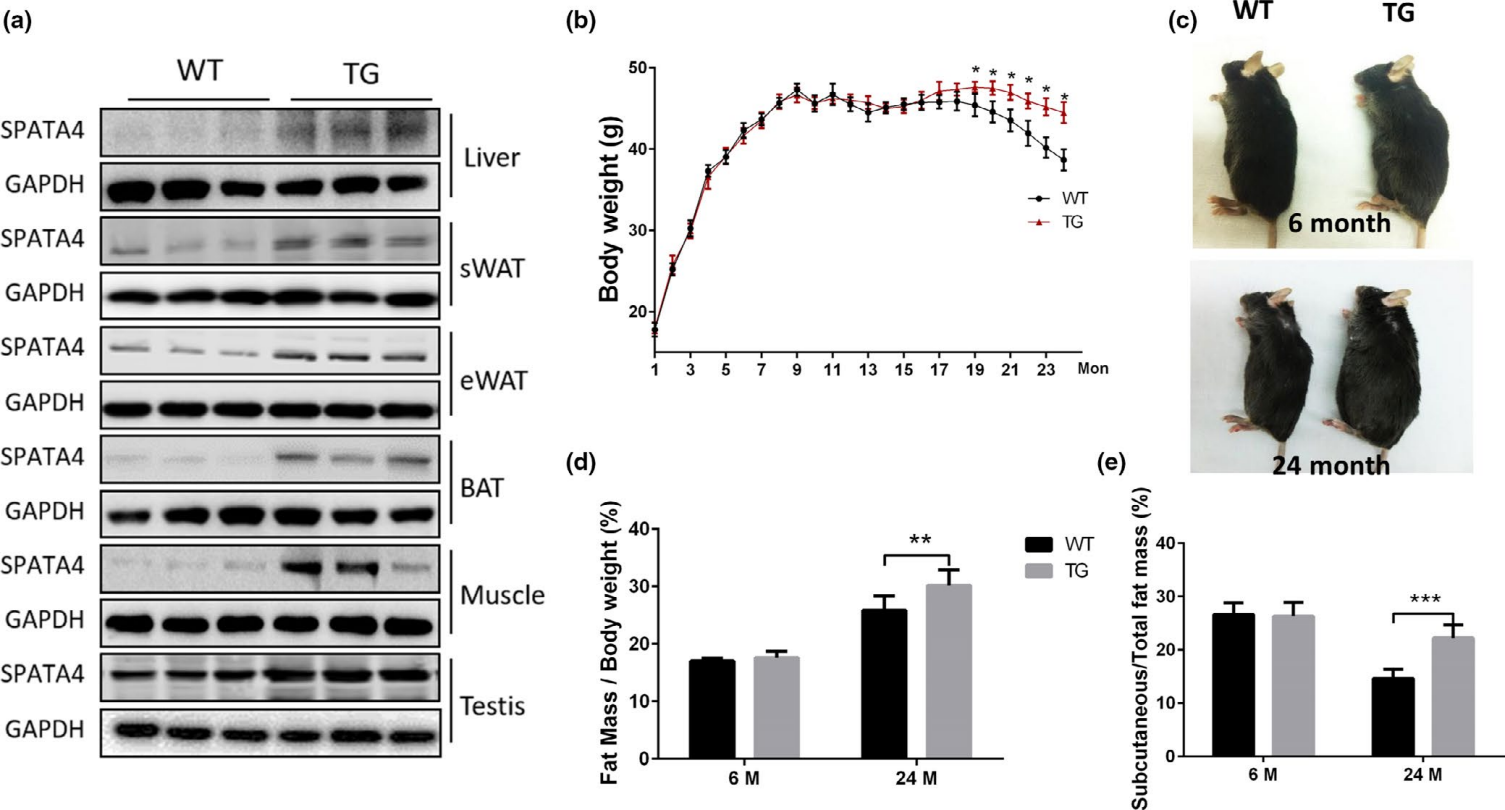
*Spata4* overexpression increased body weight and subcutaneous fat mass in old mice. (a) *Spata4* was overexpressed ubiquitously and representative Western blots show higher protein level in different organs and tissues of transgenic mice. (b) Body weight of *Spata4* transgenic mice (TG) versus wild‐type (WT) mice. *N* = 15–20, *p < 0.05. (c) Representative images of male WT and TG at 6 and 24 months. The weight ratio of fat mass to body mass (d) and the ratio of subcutaneous fat to total fat mass (e) in *Spata4* overexpressing mice and wild‐type mice. *N* = 9, **p < 0.01, ***p < 0.001

*Spata4* is highly expressed in the testes compared with other tissues (Figure S1a,b). Between male and female adipose tissue, however, there is no significant difference in the levels of *Spata4* expression (Figure S1c). We created transgenic mice ubiquitously overexpressing *Spata4* from CMV promoter and eGFP to monitor the transfection efficiency (more details in Section 2). In several tissues and organs, *Spata4* was expressed at higher levels in transgenic (TG) compared with their wild‐type (WT) littermates (Figure 1a). This overexpression was confirmed in female mice (data not shown). TG mice were born at the normal frequency, with no observed abnormalities. Under normal conditions, TG and WT mice gained weight at a similar rate until around the 19th month, when TG mice weighed more than WT mice at the same age. Specifically, the body weight of WT mice began decreasing gradually starting from 18th months of age, while it was maintained in TG mice (Figure 1b). The body size at 6 months (young) and 24 months (old) correlated with changes in body weight (Figure 1c). To further examine the causes of the observed weight differences, we measured the ratio of fat mass and lean mass to body weight and weighed the different organs and tissues from old and young, TG and WT mice. In both age groups, we found no significant differences in the weight of the heart, lung, kidney, and reproductive organs between WT and TG mice (data not shown). In the older age group, the total fat mass was significantly higher in TG mice and there was a mild but not significant increase of lean mass in TG mice compared with WT mice (Figure 1d and Table 1). To determine how the distribution of fat tissue among the different fat depots contributed to the total fat increase, we dissected and weighted different fat depots. The results showed that increased subcutaneous fat mass was the main contributor to the total increase of fat mass in old TG mice (Figure 1e and Table 1), and this difference was not seen in young mice. As *Spata4* is highly expressed in testes, to determine whether these phenotypes are contributed by SPATA4 originating from the testes, female mice were also used, and we got similar changes in female mice (Table S1). Together, our data suggest that SPATA4 gradually promotes the expansion of fat tissue, especially subcutaneous fat tissue, and attenuates aging‐induced body weight loss.

**TABLE 1:** The weight of whole body and different tissues in male mice

Weight (g)	WT 6M	TG 6M	WT 24M	TG 24M
Whole body	41.8 ± 0.54	40.9 ± 1.5	37.65 ± 1.35^b^	42.7 ± 1.59^a^
Fat mass	7.12 ± 0.11	7.2 ± 0.3	9.75 ± 0.57^b^	12.69 ± 0.41^a^, ^b^
Lean mass	31.7 ± 0.56	30.6 ± 1.32	26.6 ± 0.46	27.83 ± 1.15
Subcutaneous fat	2.27 ± 0.12	2.3 ± 0.15	1.97 ± 0.22	3.83 ± 0.22^a^, ^b^
Mesenteric fat	0.57 ± 0.03	0.77 ± 0.08	1.26 ± 0.1^b^	1.16 ± 0.06^b^
Epididymal fat	1.03 ± 0.05	0.97 ± 0.04	2.23 ± 0.29^b^	2.85 ± 0.25^b^
Perirenal fat	0.96 ± 0.08	0.90 ± 0.06	1.91 ± 0.1^b^	2.41 ± 0.18^b^
Interscapular BAT	0.78 ± 0.04	0.74 ± 0.06	1.22 ± 0.13^b^	0.99 ± 0.11
Liver	1.67 ± 0.08	1.72 ± 0.07	2.04 ± 0.11^b^	1.79 ± 0.09

The mice were dissected at 6 and 24 months of age.

^a^ *p* < 0.05, the weight was compared between wild‐type (WT) and *Spata4* transgenic mice (TG) at the same age.

^b^ *p* < 0.05, the weight was compared between 6‐ and 24‐month‐old mice with same genotype.

### 2.2 SPATA4 promotes sWAT expansion and decreases lipid accumulation in liver and brown adipose tissues

**FIGURE 2:**
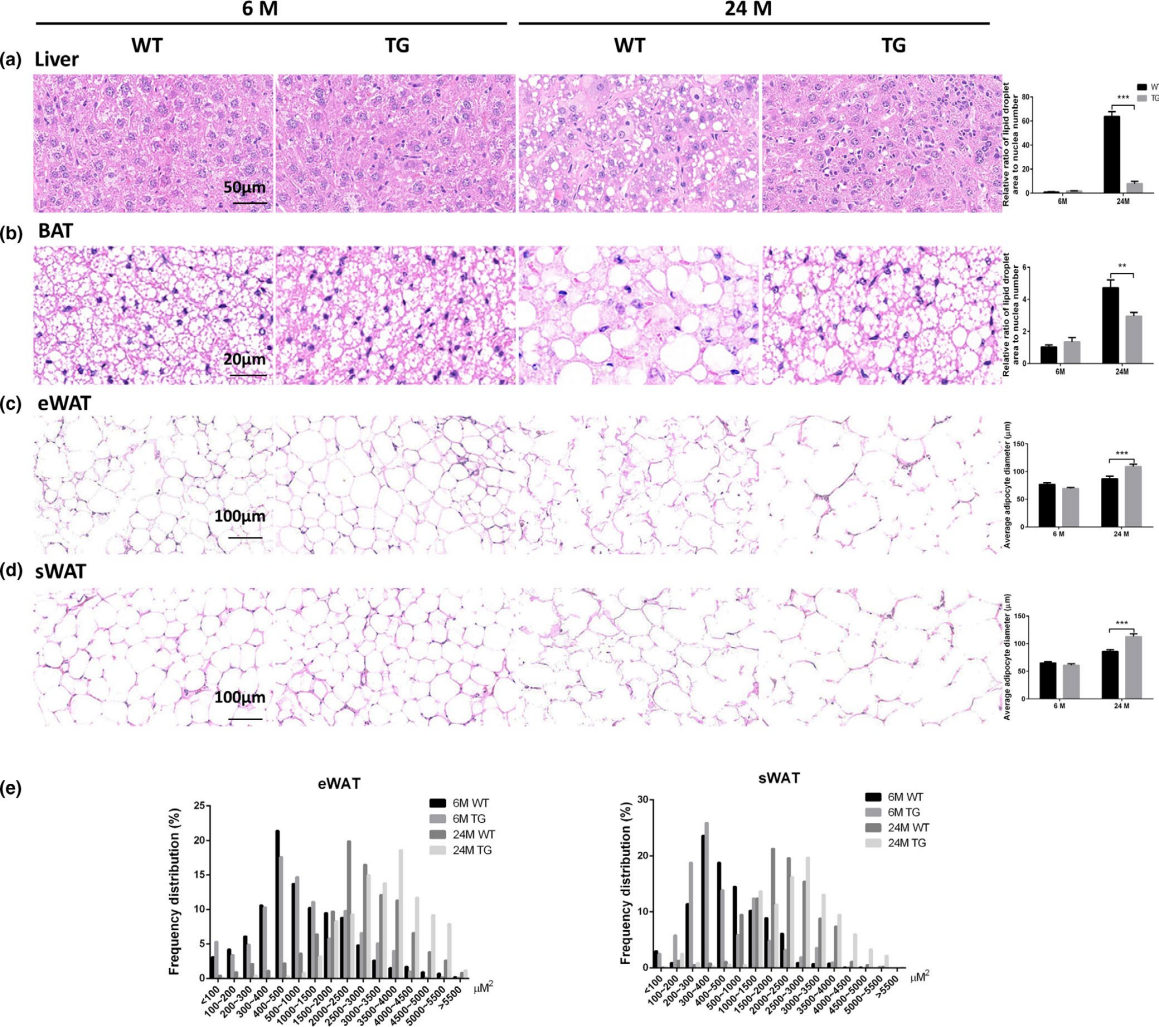
SPATA4 regulates fat distribution during aging. Histological changes were observed by H&E staining. (a) Representative images show lipid accumulation in liver tissues, and the ratio of lipid droplet area to the cell number was calculated. (b) The distribution of lipid droplets in brown adipose tissue (BAT) is shown, and the area ratio to cell number was calculated. Representative images show the morphology and diameter of adipocytes in epidydimal white adipose tissue (eWAT) (c) and subcutaneous white adipose tissue (sWAT) (d). (e) The area of each adipocyte in the view was calculated as described in the Section 2. The ratio of adipocytes within some range of area to the total adipocytes in the view was shown. *N* = 6, **p < 0.01, ***p < 0.001

Ectopic accumulation of fat is common in aged individuals, especially in the liver and brown adipose tissue (BAT) (Pararasa et al., 2015). In wild‐type mice, we found increases in liver weight and BAT weight at an old age compared with a young age. Intriguingly, this increase of liver weight in old mice was almost eliminated by *Spata4* overexpression. The difference in BAT weight between old and young mice was also decreased in the TG group (Table 1). We performed histological tests to further look for structural changes in the liver and BAT that may underlie these weight differences. H&E staining showed more lipid droplet accumulation in liver and BAT tissues of WT aged mice compared with the young mice, and these aging‐induced changes were significantly attenuated in TG old mice (Figure 2a,b, Figure S2a,b). These data suggest that there is less ectopic fat accumulation in TG old mice, which might explain the lower weight of liver and BAT tissues compared with WT old mice.

As fewer lipid droplets accumulated in liver and BAT tissues of TG old mice, we reasoned that lipid may be accumulating in a different location. To investigate this, we examined histological changes in the fat tissues of different regions. Fat tissue at different regions varies in function. Subcutaneous adipose tissue plays a more beneficial role in maintaining metabolic homeostasis than visceral adipose tissue (Gulcelik et al., 2013). Abdominal subcutaneous white adipose tissue (sWAT) and epididymal white adipose tissue (eWAT) were examined histologically to represent subcutaneous and visceral adipose tissues respectively. Intriguingly, the adipocytes in WT old mice showed a more irregular morphology than the adipocytes in TG old mice (Figure 2c,d, Figure S2c,d), indicative of adipocyte damage, accompanied by higher inflammation (Gentile et al., 2019; Lee et al., 2015). The cell sizes of adipocytes in both sWAT and eWAT were obviously increased in both WT and TG aged mice compared with young mice. However, the adipocyte cell size in TG old mice was even larger than the ones from WT old mice, suggesting increased accumulation of lipid droplets, which might partially explain the decreased accumulation of lipid droplets in liver and BAT tissue (Figure 2c–e and Figure S2c–e), although there was no significant difference in eWAT weight between WT and TG old mice (Table 1). These data above suggest SPATA4 promotes lipid droplet accumulation in WAT.

### 2.3 SPATA4 maintains serum triglyceride and stimulates lipid oxidation in aged mice

**FIGURE 3:**
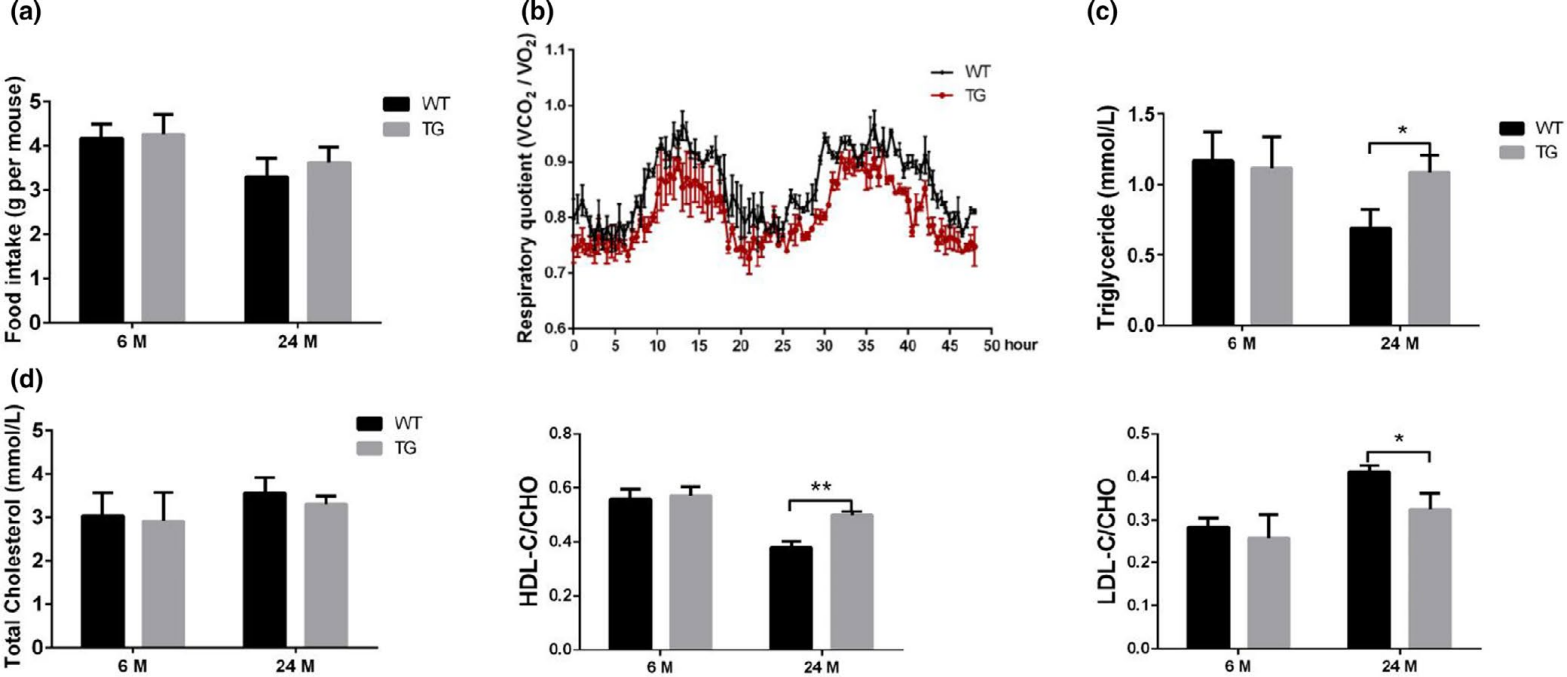
The effect of SPATA4 on lipid oxidation and serum lipid level. (a) Food intake was measured at 6 and 24 months of age. *N* = 9. (b) The respiratory quotient (V_CO2_/V_O2_) during 48 h in WT and TG old mice was determined by means of metabolic chambers. *N* = 3. (c) The serum levels of the triglyceride were measured. *N* = 6. (d) The serum level of the total cholesterol, the ratio of high‐density lipoprotein cholesterol (HDL‐C), and low‐density lipoprotein cholesterol (LDL‐C) to total cholesterol (CHO) were shown. *N* = 6, *p < 0.05, **p < 0.01

To study whether the food intake contributes to differential fat mass, we measured the level of food intake by young and old, WT and TG mice. There was no significant difference induced by *Spata4* overexpression neither at young nor old age (Figure 3a). TG old mice showed a mildly lower level of respiratory quotient than WT old mice, which suggests increased lipid oxidation (Figure 3b). As for serum lipid level, we found triglyceride level was increased in old TG mice compared with old WT mice, but this difference was not observed in total cholesterol level (Figure 3c,d). Further tests showed that the ratio of high‐density lipoprotein cholesterol level to total cholesterol level increased in TG old mice, concomitant with a decreased ratio of low‐density lipoprotein to total cholesterol (Figure 3d). Combined with our observation of increased fat storage, these data suggest that SPATA4 enhances lipid metabolism.

### 2.4 SPATA4 attenuates inflammation and apoptosis and enhances insulin sensitivity in fat tissues of aged mice

**FIGURE 4:**
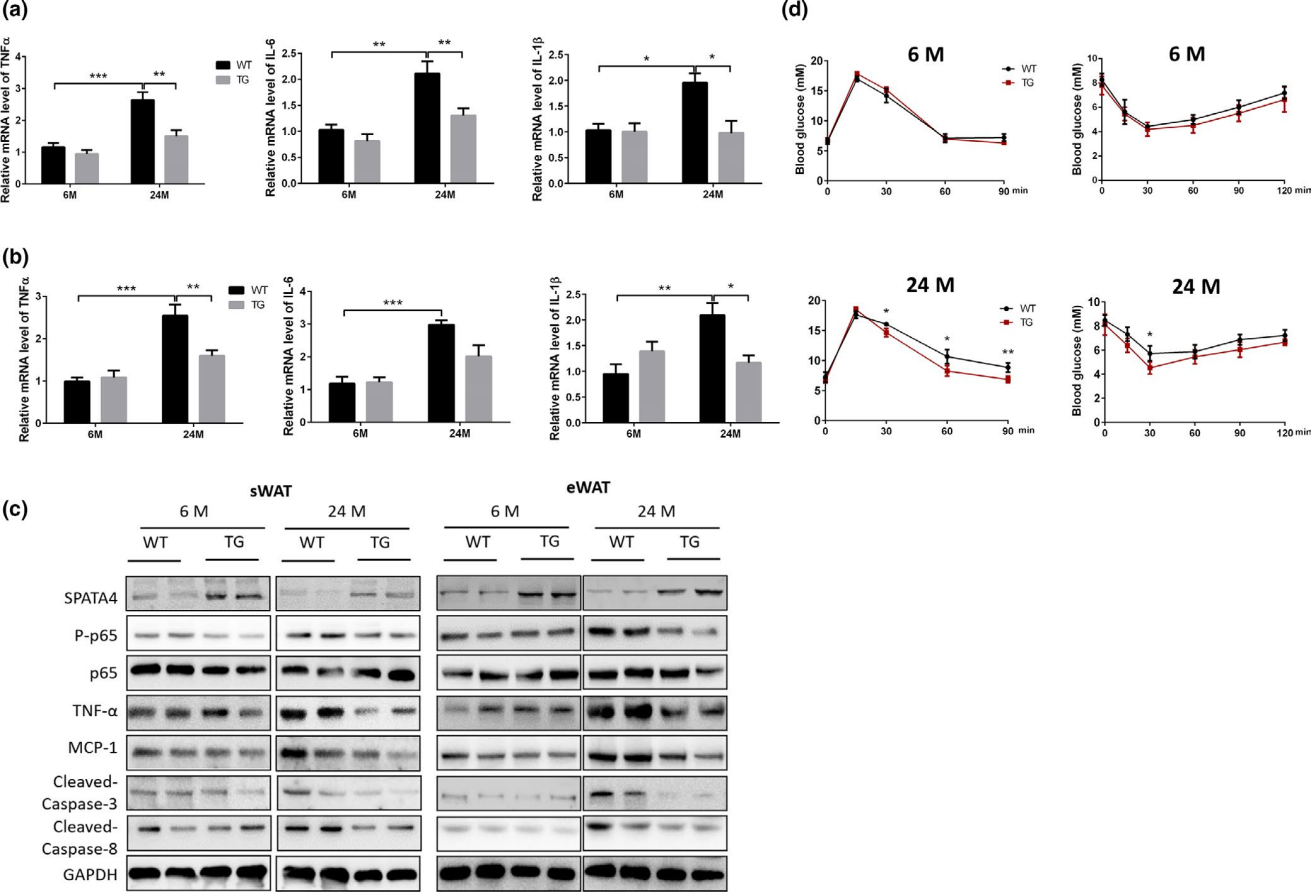
The effect of SPATA4 on the inflammation of fat tissue and insulin sensitivity. The mRNA levels of inflammatory factors in subcutaneous white adipose tissue (sWAT) (a) and epidydimal white adipose tissue (eWAT) (b) were determined by qPCR. *N* = 6, *p < 0.05, **p < 0.01, ***p < 0.001. (c) Representative blots showed the protein levels of inflammation and apoptosis‐related proteins. (d) The insulin sensitivity of WT and TG male mice was determined by a glucose tolerance test (left) and an insulin tolerance test (right). *N* = 6, *p < 0.05, **p < 0.01

It has been reported that chronic inflammation is closely correlated with various aging‐related diseases, including diabetes and cancer (Gilbert & Slingerland, 2013; Pararasa et al., 2015). WAT has been shown to be a primary location where inflammation and insulin resistance become established in aged animals (Wu & Ballantyne, 2017; Yazici & Sezer, 2017). To explore the effect of SPATA4 on inflammation in fat tissues, we examined the expression of typical inflammatory factors in both sWAT and eWAT from male mice, as SPATA4 increased the WAT mass significantly in old mice. The mRNA levels of tumor necrosis factor α (TNF‐α), interleukin 6 (IL‐6), and interleukin 1β (IL‐1β) in sWAT were decreased in old TG mice compared with old WT mice (Figure 4a). We also obtained similar results in eWAT, except for IL‐6 (Figure 4b). These results got confirmed in female mice (Figure S3a,b). Intriguingly, there was a mild decrease of SPATA4 in TG old mice versus TG young mice rather than in WT mice. NF‐κB is a key regulator of the senescence‐associated secretory phenotype (SASP), and the phosphorylation of its subunit p65 at Ser536 induces its activation. p65 phosphorylation was significantly lower in TG old mice than in WT old mice. The protein level of TNF‐α further reflected the change in mRNA level. We then measured expression of monocyte chemoattractant protein 1 (MCP‐1) to evaluate macrophage recruitment. The protein level of MCP‐1 was lower in both eWAT and sWAT of old TG mice than old WT mice (Figure 4c and Figure S3c,d). TNF‐α can activate caspase‐mediated cell apoptosis and irregular morphology, suggesting the occurrence of cell apoptosis in the WAT of WT old mice. The protein levels of cleaved‐caspase3 and caspase8 were both decreased in the eWAT of old TG mice, suggesting decreased apoptosis in eWAT (Figure 4c and Figure S3c,d).

Adipose tissue inflammation is closely related to decreased insulin sensitivity (Olefsky & Glass, 2010). We observed an improved glucose tolerance in old TG mice, suggesting higher insulin sensitivity, and we also found a faster and stronger change of glucose level after injection of insulin in old TG mice compared with old WT mice. However, the difference was not observed at young age (Figure 4d and Figure S3e). These data indicate that SPATA4 maintains the homeostasis of fat tissue by inhibiting inflammation and apoptosis.

### 2.5 SPATA4 promotes preadipocyte differentiation

**FIGURE 5:**
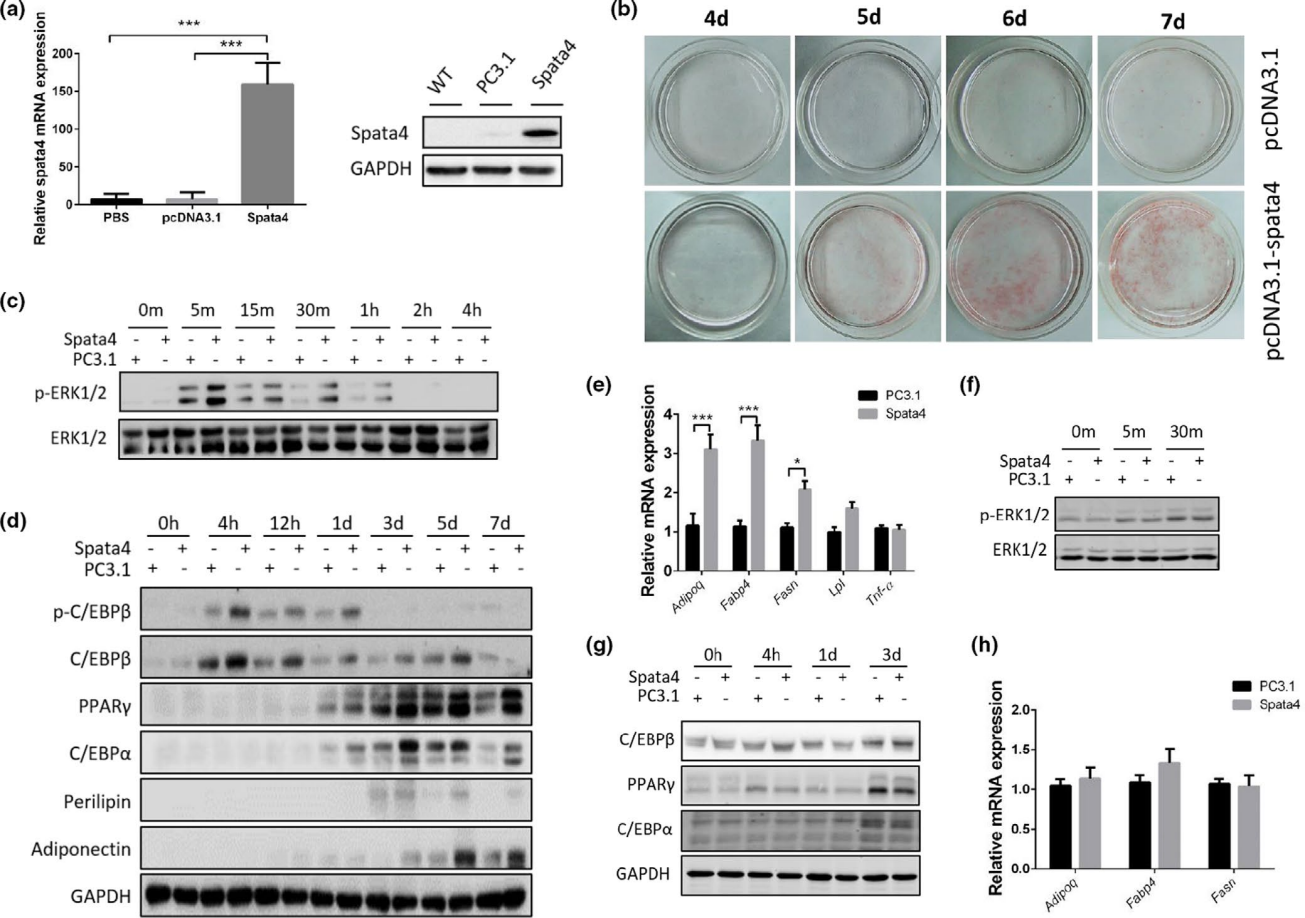
The effect of SPATA4 on adipocyte differentiation. (a) *Spata4* was transfected into 3T3‐L1 cells through pcDNA3.1 /myc‐His A plus vector and an empty vector (PC3.1) was used as a control. The overexpression of *Spata4* was confirmed by qPCR and Western blot. (b) The differentiation of 3T3‐L1 cells was induced as described in the methods section. Oil Red O staining was used to monitor the lipogenesis. Representative images of differentiating cells at 4–7 days were compared between *Spata4* transgenic cells and control cells. (c) Western blots show the phosphorylation of ERK1/2 within 4 h after induction of differentiation in 3T3‐L1 cells. (d) The levels of adipocyte differentiation‐related protein after induction are shown in Western blots. (e) The mRNA levels of *Adipoq*, *Fabp4*, *Fasn*, *Lpl*, and *Tnfα* at the 7th day after induction are compared between *Spata4* transgenic and control cells. *N* = 4, *p < 0.05, **p < 0.01. (f) U0126 was used to inhibit ERK1/2 phosphorylation, and representative blots showed the phosphorylation levels after induction of differentiation. (g) Under U0126 treatment, the protein level of adipocyte differentiation‐related‐genes is shown at different time points after induction. (h) Under U0126 treatment, the mRNA levels of *Adipoq*, *Fabp4*, and *Fasn* at the 7th day after induction were compared between *Spata4* transgenic and control cells

To determine the mechanism of how SPATA4 functions in fat tissue, we overexpressed *Spata4* in preadipocytes (3T3L1) and looked for molecular changes. mRNA and protein levels confirmed the overexpression of *Spata4* in 3T3L1 cells (Figure 5a). We did not see any changes in cell proliferation under normal conditions (data not shown). During the induction of differentiation into mature adipocytes, Oil Red O staining showed more positive signals in *Spata4* transfected cells starting from the fourth day, which indicated increased lipogenesis in the cells (Figure 5b). ERK1/2 activation by phosphorylation is reported to be an essential step for adipocyte differentiation (Prusty et al., 2002). We found that ERK1/2 was phosphorylated at 5 min after differentiation induction and the activation persisted for 1 h. The ratio of phosphorylated ERK1/2 to total ERK1/2 was higher in *Spata4* transfected cells (Figure 5c). A cascade involving CCAAT‐enhancer‐binding proteins (C/EBPs) and peroxisome proliferator‐activated receptor γ (PPARγ) orchestrates the adipogenesis process (Tang & Lane, 2012). The phosphorylation of C/EBPβ was higher in *Spata4* transfected cells, concomitant with increased expression of downstream PPARγ and C/EBPα. The protein levels of Perilipin and Adiponectin were both higher in *Spata4* transfected cells at different time points (Figure 5d). The fatty‐acid‐binding proteins (FABPs) have been reported to facilitate the transfer of fatty acids between extra‐ and intracellular membranes, and *Fabp4* is expressed in mature adipocytes. The mRNA level of *Fabp4* was significantly higher in *Spata4* transfected cells. We also found an increase in *Fasn* mRNA level, suggesting enhanced fatty acid synthesis (Figure 5e). To determine whether SPATA4 functions by promoting ERK1/2 activation, we pretreated the cells with a MEK inhibitor to inhibit the phosphorylation of ERK1/2 (Figure 5f). After inhibition of ERK activation, there was no difference of adipocyte differentiation‐related protein levels between control and *Spata4* transfected cells (Figure 5g). Furthermore, *Spata4* overexpression did not increase expression of *Adipoq*, *Fabp4* or *Fasn* after inhibition of ERK activation (Figure 5h). These data suggest SPATA4 promotes differentiation of preadipocyte in an ERK‐dependent way.

**FIGURE 6:**
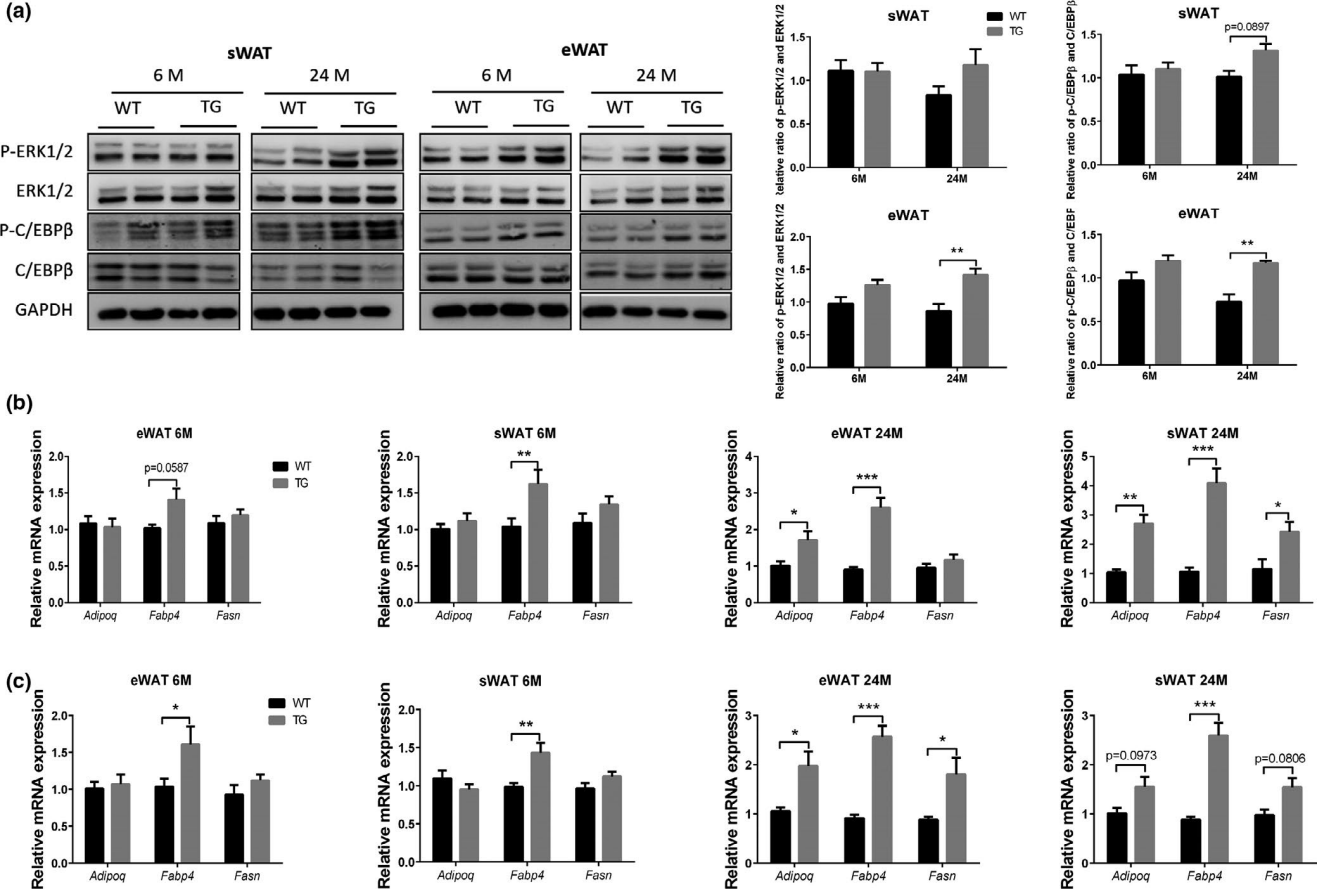
The effect of SPATA4 on ERK signaling and adipocyte differentiation in adipose tissue. (a) The phosphorylation of ERK1/2 and C/EBPβ was measured in subcutaneous white adipose tissue (sWAT) and epidydimal white adipose tissue (eWAT). Representative blots are shown. *N* = 4, **p < 0.01. qPCR was used to determine the expression of *Adipoq*, *Fabp4* and *Fasn* in sWAT and eWAT from male (b) and female (c) mice. *N* = 4–6, *p < 0.05, **p < 0.01, ***p < 0.001

To further study the mechanism of SPATA4 in vivo, we measured the phosphorylation of ERK1/2 and C/EBPβ in adipose tissues of TG and WT mice. SPATA4 increased the phosphorylation of ERK1/2 and C/EBPβ in eWAT of old mice and there was a mild but not significant increase in sWAT (Figure 6a). SPATA4 increased the expression of *Fabp4* and *Adipoq* in both sWAT and eWAT of old TG mice and *Fasn* only in sWAT. Intriguingly, the expression *Fabp4* was increased by SPATA4 even at a young age, suggesting that the expression of *Fabp4* is strongly regulated by SPATA4 (Figure 6b). To explore whether the effect of SPATA4 is gender‐dependent, we also measured the expression of genes in the adipose tissue of female mice, and found similar results (Figure 6c). All in all, these data indicate that SPATA4 promotes the differentiation of preadipocytes and regulates fatty acid transfer.

## 3 DISCUSSION

We have previously reported that SPATA4 could promote osteoblast differentiation through the ERK‐activated RunX2 pathway (Wang et al., 2011) and protect Hela cells from etoposide‐induced apoptosis (Jiang et al., 2015). However, the specific mechanism and the biological function of SPATA4 in vivo have still not been described. To explore these questions, we made *Spata4* mutant mice. Unexpectedly, *Spata4* deficiency did not cause any obvious changes to the mice, Neither *Spata4* knockdown nor overexpression affected reproductivity, despite *Spata4* being highly expressed in the testis. Intriguingly, *Spata4* overexpression mice showed increased lipid accumulation in fat tissue and enhanced lipid metabolism, a phenotype that was observed only in old but not young mice and these effects are consistent in male and female mice.

Obesity has long been understood to be a risk factor for various metabolic dysfunctions, like insulin resistance and dyslipidemia. In obesity, increased body weight usually results from more fat mass accumulation (Despres & Lemieux, 2006). Because fat tissues in different regions vary in size and function and these tissues are associated with differential cellular compositions and cytokine secretion profiles, and the proportional distribution of regional fat tissues is relevant in many metabolic diseases (Tchkonia et al., 2013). The WAT has become increasingly recognized as an important endocrine organ that secretes a number of biologically active “adipokines” (Lazar, 2006; Zhang et al., 1994). Visceral WAT is more involved in the development of metabolic diseases than subcutaneous WAT (Gulcelik et al., 2013). Aging induces redistribution of fat tissue defined by a decreased peripheral subcutaneous adiposity and increased visceral adiposity, which exacerbates metabolic dysfunction. As aging progresses, the uptake of fatty acids and lipids declines, resulting in ectopic accumulation of lipids in liver and other organs and tissues (Johannsen et al., 2012; Stahl et al., 2020; Yamamoto et al., 2014). Under the same level of food intake, TG old mice showed increased subcutaneous fat accumulation manifested by larger lipid droplets. At the same time, aging‐induced fatty liver and ectopic lipid deposition in the BAT were significantly improved in TG old mice, suggesting that SPATA4 promotes lipid uptake by adipocytes and deductively prohibits the accumulation of lipids in the liver and BAT. The transcriptional coactivator Cidea has been reported to improve metabolic profile by expanding adipose tissue (Abreu‐Vieira et al., 2015). These data also indicate that aging‐accompanied obesity should be interpreted by fat accumulation at different locations. TG old mice showed increased mass in sWAT, but not other depots of WAT, and the expression of inflammatory cytokines and *Fasn* in sWAT and eWAT were also differently affected by *Spata4* overexpression, supporting the notion that regional fat tissues vary in function. The mechanism of how SPATA4 functions specifically will require further investigation.

Aging is usually accompanied by sustained fat‐tissue immune‐response activation, pro‐inflammatory cytokine release, impaired insulin responsiveness, and increased lipolysis, which is partially caused by chronic inflammation (van der Kallen & van Greevenbroek, 2010; Tchkonia et al., 2010). The pro‐inflammatory response of adipose tissue manifests a shift from storing to releasing fatty acids (Faty et al., 2012; Zhang et al., 1996). In addition to their role as major sources of cellular fuel, fatty acids can serve as signaling molecules exerting adverse effects on cell function, including interference with insulin signaling (Li et al., 2020). The mRNA levels of inflammatory cytokines as well as activation of NF‐κB was decreased in the WAT of TG old mice compared with WT old mice. There was no difference at SAβ‐gal staining of WAT between TG and WT old mice (data not shown), and *Spata4* overexpression in preadipocytes did not change the activation of NF‐κB, expression of TNF‐α or SAβ‐gal staining, indicating that SPATA4 is not a direct regulator of cell senescence or inflammation. Decreased inflammation also attenuated cell apoptosis and improved morphology of adipocytes in WAT. We speculate that the improvement of insulin sensitivity in TG old mice was due to improved fatty liver and well‐functioning WAT. Furthermore, increased adiponectin is associated with better insulin sensitivity (Yadav et al., 2013).

ERK1/2 signaling is essential for adipocyte differentiation (Prusty et al., 2002). Activated ERK1/2 can phosphorylate C/EBPβ, which is a transcriptional factor regulating the expression of PPARγ and C/EBPα. We have previously found SPATA4 can bind to ERK1/2 and enhance its phosphorylation, promoting osteoblast differentiation (Wang et al., 2011). Here, we reported that SPATA4 promotes the phosphorylation of ERK1/2 in preadipocytes, inducing subsequent activation of C/EBPβ and increased expression of PPARγ and C/EBPα, contributing to a faster differentiation defined by increased expression of Perilipin and Adiponectin and increased accumulation of lipid droplets as measured by Oil Red O staining. PPARγ is predominantly expressed in adipose tissues, both white and brown, where it plays an important anabolic role in facilitating fat storage, adipogenesis, and thermogenesis (Ferre, 2004; Siersbaek et al., 2010). PPARγ also contributes to the homeostasis of adipose tissue under challenging physiological circumstances such as aging (Corrales et al., 2018). In contrast to the expression of adipokines such as TNF‐α and MCP‐1, which causes insulin resistance, Adiponectin expression is reduced in obese, insulin‐resistant rodent models (Hu et al., 1996). SPATA4 increased the protein levels of PPARγ and Adiponectin in adipocytes, which might partially explain the beneficial effects of SPATA4 on insulin sensitivity in vivo. Fatty‐acid‐binding proteins (FABPs) are a family of lipid chaperones contributing to systemic metabolic regulation via several lipid signaling pathways (Smathers & Petersen, 2011). Fatty‐acid‐binding protein 4 (FABP4) is mainly expressed in adipocytes and macrophages and plays important roles in the development of insulin resistance and inflammation (Furuhashi, 2019; Furuhashi et al., 2014). SPATA4 enhances the expression of *Fabp4* significantly both in preadipocytes and WAT, and this difference in expression was observed even at a young age, indicating regulation of FABP4 could be a key mechanism by which SPATA4 promotes lipid accumulation in WAT.

*Spata4* was overexpressed ubiquitously in mice, but we found that only fat tissue was affected significantly, compared with other tissues and organs. One possible explanation for this is that SPATA4 may bind with certain adipocyte‐specific proteins to regulate the activity of some signaling pathways, a hypothesis that warrants further research. Another interesting phenomenon is that the difference between WT and TG mice is only apparent at an older age, and not in younger mice. This age‐dependent effect of gene mutation has been also found in other studies (Schwer et al., 2010). We speculate that the difference can only be visible under some stress or adverse environment, as damage accumulates in the aged mice resulting from various exogenous and endogenous stresses. It is also possible that SPATA4 is involved in some aging‐related signaling pathways, which are not active or active only at a low level in young mice.

In this study, we overexpressed *Spata4* in preadipocytes and ubiquitously in mice to conclude that 1) SPATA4 promotes preadipocyte differentiation through activation of ERK1/2 and C/EBPβ pathway, and 2) in vivo, SPATA4 attenuates aging‐induced ectopic distribution of fat and improves metabolic homeostasis. Understanding the signaling mechanism of SPATA4 function in these processes will help clarify effective targets improving aging‐induced dysfunctional fat metabolism.

## 4 METHODS AND MATERIALS

### 4.1 Generation of transgenic mice and mouse feeding

*Spata4* transgenic mice were generated in the C57BL/6 mouse strain according to the standard procedure of Cyagen Transgenic Animal Center (Suzhou, China). cDNA was amplified from a plasmid encoding mouse *Spata4*, which was subcloned into the CMV promoter vector with eGFP. At 1–2 weeks of age, tail DNA was analyzed to confirm mice positive for the transgene. The following primers were used for genotyping: forward, 5′‐ACGTAAACGGCCACAAGTTC‐3′; reverse, 5′‐GATCTTGAAGTTCACCTTGATGC‐3′. The transgenic PCR product size is 440 bp. The male transgenic mice were compared with wild‐type littermates at 6 months and 24 months of age respectively. Body weight was measured every 2 weeks. The mice were dissected after blood collection by heart puncture and organs, and tissues were weighed. Mice were housed in cages with free access to food and water in a temperature‐controlled room with a 12‐h light/dark cycle. The mice were specific‐pathogen‐free (SPF) grade animals and were fed in a barrier SPF environment. All animal experimental protocols were approved by Institutional Animal Care and Use Committee (IACUC) of Tsinghua University.

### 4.2 Cell culture

3T3‐L1 preadipocytes cells were cultured in high‐glucose DMEM with 10% FBS (Thermo) and penicillin/streptomycin (100 U/ml) (Thermo) at 37°C with 5% CO_2_. pcDNA3.1/myc‐His A plus vector (Invitrogen) was used to carry *Spata4* into the cells through electroporation (Takara) according to the manufacturer's procedure into 3T3‐L1 preadipocytes and empty pcDNA3.1 vector was used as a control. G418 was used to select and maintain *Spata4* overexpressing cells. During differentiation, 3T3‐L1 cells were cultured to confluency for 48 h and then incubated in differentiation medium consisting of insulin (10 μg/ml) (Solarbio), dexamethasone (0.25 μM) (DEX, Sigma‐Aldrich), 3‐isobutyl‐1‐methylxanthine (0.25 mM) (IBMX, Sigma‐Aldrich). Two days later, the medium was switched to DMEM consisting of 10 μg/ml insulin and changed every other day till full differentiation. For experiments performed with U0126 (5 μM) (Cell Signaling Technology), post‐confluent 3T3‐L1 preadipocytes were preincubated for 30 min before the induction of differentiation. All cells were negative for mycoplasma contamination before use.

### 4.3 Western blotting

Tissue and cell samples were prepared in middle RIPA lysis buffer (Biomiga), supplemented with a cocktail of protease (AbMole Bioscience) and phosphatase inhibitors (Solarbio). Western blotting was performed as described previously before (Li et al., 2020). An equal amount of protein was run on 10% SDA‐PAGE and then transferred to a polyvinylidene difluoride (PVDF) membrane (Millipore). The membrane was blocked for 24 min in 5% skim milk (OXOID) containing 0.1% fetal bovine serum (Gibco). Blocked membranes were incubated overnight at 4°C with primary antibody diluent. The blots were then incubated with the appropriate horseradish peroxidase (HRP)‐conjugated secondary antibody (Abcam) diluent at room temperature for 2 h. Antibody bound protein was detected by SuperSignal West Pico Chemiluminescent Substrate (Thermo). Protein signal was visualized using a Chemi Capture (CLINX) and bind intensity was analyzed by ImageJ software and normalized. Primary antibodies against the proteins were as follow: SPATA4 (#ab153715, Abcam), GAPDH (#5174, Cell Signaling Technology), NF‐κB p65 (#8242, Cell Signaling Technology), NF‐κB p‐p65 (#3033, Cell Signaling Technology), TNFα (#3707, Cell Signaling Technology), MCP1 (#ab25124, Abcam), Cleaved caspase‐3 (#9664, Cell Signaling Technology), Cleaved caspase‐8 (#8592, Cell Signaling Technology), ERK1/2 (#9102, Cell Signaling Technology), p‐ERK1/2 (#4377, Cell Signaling Technology), C/EBPβ (#2487, Cell Signaling Technology), p‐C/EBPβ (#2484, Cell Signaling Technology), C/EBPα (#8178, Cell Signaling Technology), PPARγ (#2435, Cell Signaling Technology), Perilipin (#9349, Cell Signaling Technology), Adiponectin (#2789, Cell Signaling Technology). Second antibodies: Goat anti‐rabbit IgG and goat anti‐mouse IgG (Santa Cruz Biotechnology).

### 4.4 Quantitative RT‐PCR (QPCR) assay

RNA was extracted from cells or adipose tissues using Trizol LS Reagent (Invitrogen) and purified using RNAEasy mini kit (Qiagen). A total of 800 ng of RNA for each sample was reverse transcribed using the First Strand cDNA synthesis kit (TOYOBO). The resulting cDNA was used as template for qPCR analysis with SYBR Green QPCR Master Mix (TOYOBO). Primers were purchased from Invitrogen. Gene quantification was performed in triplicate on the CFX96TM Real‐Time System (BIO‐RAD). Dates were calculated using the ΔΔCt method. Primers were shown in Table S2.

### 4.5 H&E staining

All tissues were fixed in 4% paraformaldehyde for a day at 4°C and then embedded in paraffin. 4 μm sections were dehydrated and stained with hematoxylin and eosin (H&E) according to the manufacturer's instructions (Servicebio). Images were captured by a light microscope (Leica), and the area of the lipid droplet was quantified by software Zen 2.3 (blue edition, Carl Zeiss Microscopy GmbH, 2011) in a blinded manner. The analysis pipeline mainly included class, frame, automatic segmentation, interactive segmentation, and statistics. Class was used to set up analyze channels and the color of selected objects. Next, the region of interest was framed using the rectangle selection tool. Then, the RGB values of the boundary of lipid droplet or adipocyte were detected by color picker. These values were regarded as threshold values, and the region whose RGB value beyond the threshold value would be selected. For some regions that were unable to be recognized by the software, we used an interactive segmentation tool to fill the unselected region or to discard the over‐selected region.

### 4.6 Metabolic assessment

The metabolic rate of the mice was measured in metabolic chambers (PhenoMaster TSE system) at the Laboratory Animal Research Center of Tsinghua University. 24‐month‐old age mice were housed individually. Light and feeding conditions were kept the same as in the home cages. All the mice were acclimated to the monitoring cages for 24 h before recording. The concentration of O_2_ and CO_2_ was measured by calculating the air entering and leaving the chambers, and the respiratory exchange ratio is the ratio of CO_2_ production and O_2_ consumption.

### 4.7 Plasma analysis

Blood was centrifugated at 1,500 g for 10 min at 4°C to isolate plasma. The plasma was stored at −80°C until thawing for further use. Plasma levels of triglycerides, cholesterol, high‐density lipoprotein cholesterol, and low‐density lipoprotein cholesterol were assayed by automatic biochemical immunity analyzer (COBAS 6000, Roche).

### 4.8 GTT and ITT

For glucose tolerance test (GTT), mice were fasted for 16 h and then injected intraperitoneally with d‐glucose (2 g/kg body weight). Glucose concentration was determined using a One Touch glucometer (Roche) in the tail vein at 0, 15, 30, 60, and 120 min. For insulin tolerance test (ITT), mice were fasted for 4 h and then injected intraperitoneally with insulin (0.75 U/kg body weight) (Solarbio) and blood glucose was measured at 0, 15, 30, 60 min using a glucometer.

### 4.9 Oil Red O staining

Endo‐cellular lipid accumulation was determined by Oil Red O staining according to the manufacturer's instructions (Solarbio). In brief, 3T3‐L1 cells, after adipogenic induction, were washed with phosphate‐buffered saline and fixed with 4% paraformaldehyde for 30 min. After two washes with distilled water, cells were dyed with 0.5% ORO stain for 20 min at room temperature after washed with 60% isopropanol. The cells stained with ORO were washed five times to remove the excess dye and were observed and photographed under the microscope.

### 4.10 Statistical analysis

All the results are expressed as means ± SEM. Comparisons between two groups were performed using an unpaired, two‐tailed Student's *t* test, and two‐way ANOVA was used for multiple group comparison. Data were analyzed in Graph Pad Prism 6.0 software.

## CONFLICT OF INTEREST

Authors declare no competing interests.

## AUTHORS' CONTRIBUTIONS

Z.C.L. and Z.W. conceived the study; Z.C.L, K.X., and Z.W. designed the experiments; Z.C.L. and K.X. conducted most of the experiments and data analyses; S.Z. and Y.N.G. performed animal feeding, dissection, and tissue staining. Y.N.G. and H.L.C. conducted staining analyses and quantification; Q.F.L., J.Q.N., and Z.W. contributed to the discussion and data interpretation; Z.C.L and K.X. wrote the manuscript.

## Supporting information

Fig S1

Fig S2

Fig S3

Table S1

Table S2

## Data Availability

All data are available in the manuscript or the supplementary materials. Correspondence and requests for materials should be addressed to corresponding author Z.W.
