# Supplementary figures and images for: SPATA4 improves aging‐induced metabolic dysfunction through promotion of preadipocyte differentiation and adipose tissue expansion
Source: Aging Cell. 2020 Dec 13;20(1):e13282. doi: 10.1111/acel.13282 (PMC7811838; doi:10.1111/acel.13282)

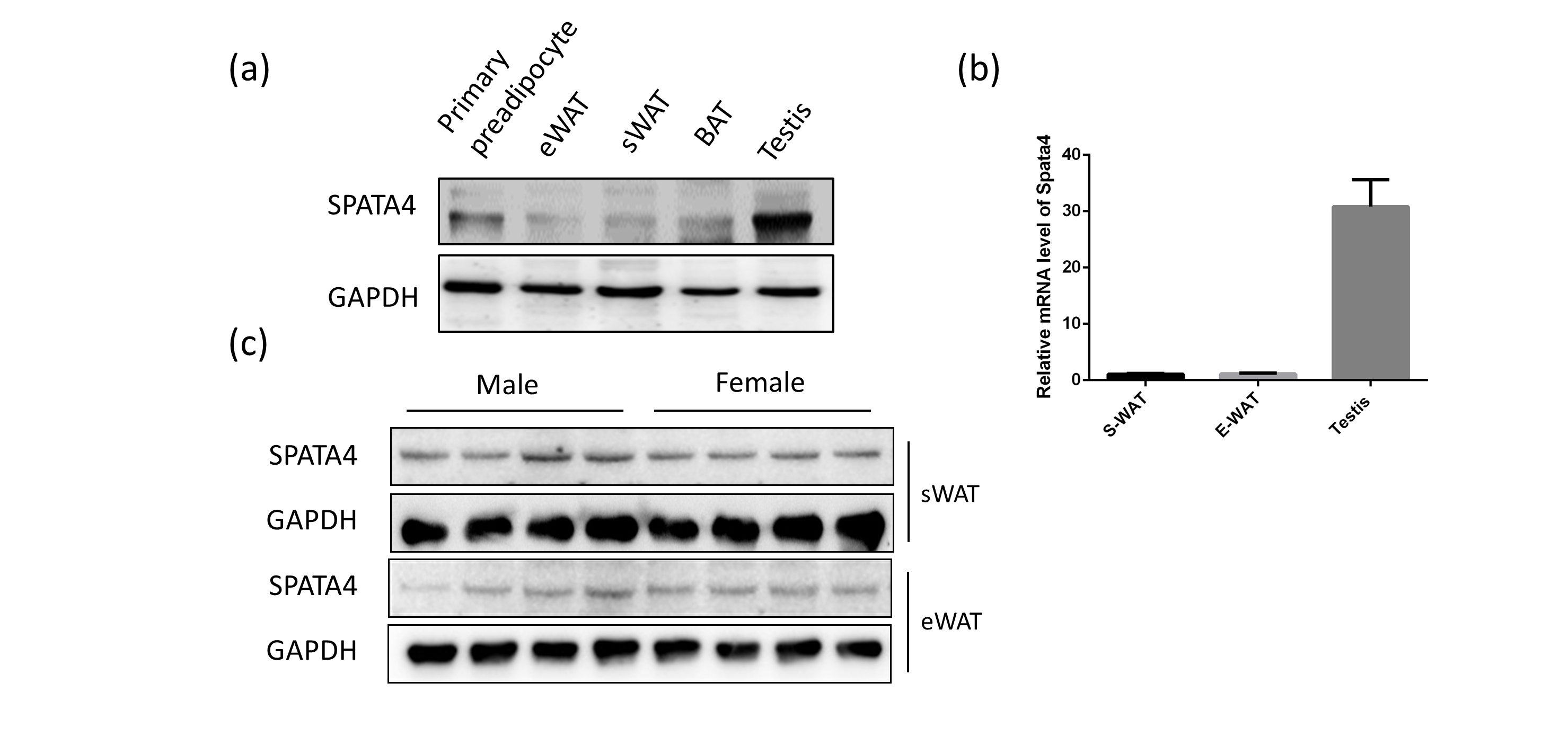

Supplement: Supplementary file 1 — Fig S1 [file ACEL-20-e13282-s001.tif]

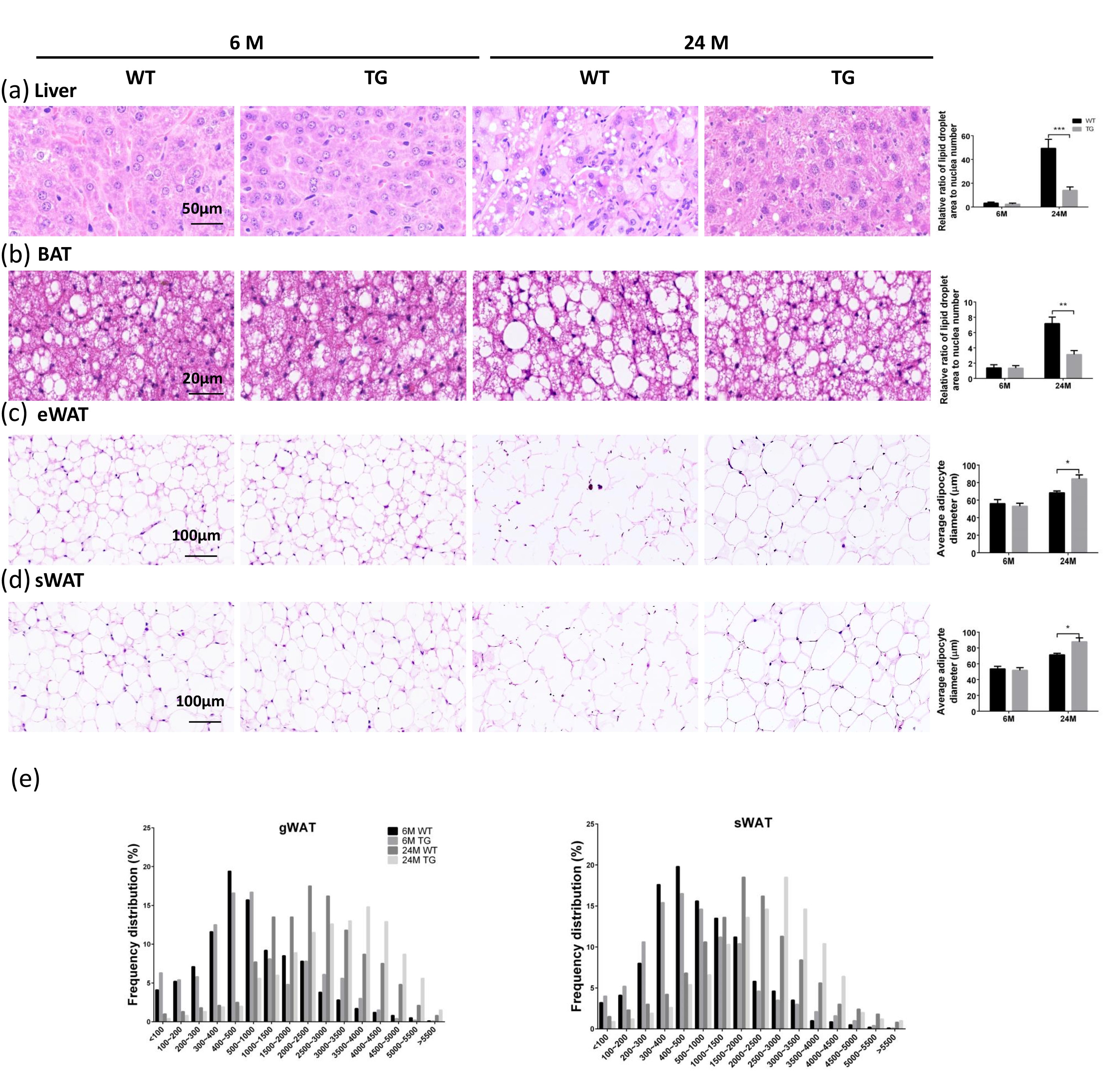

Supplement: Supplementary file 2 — Fig S2 [file ACEL-20-e13282-s002.tif]

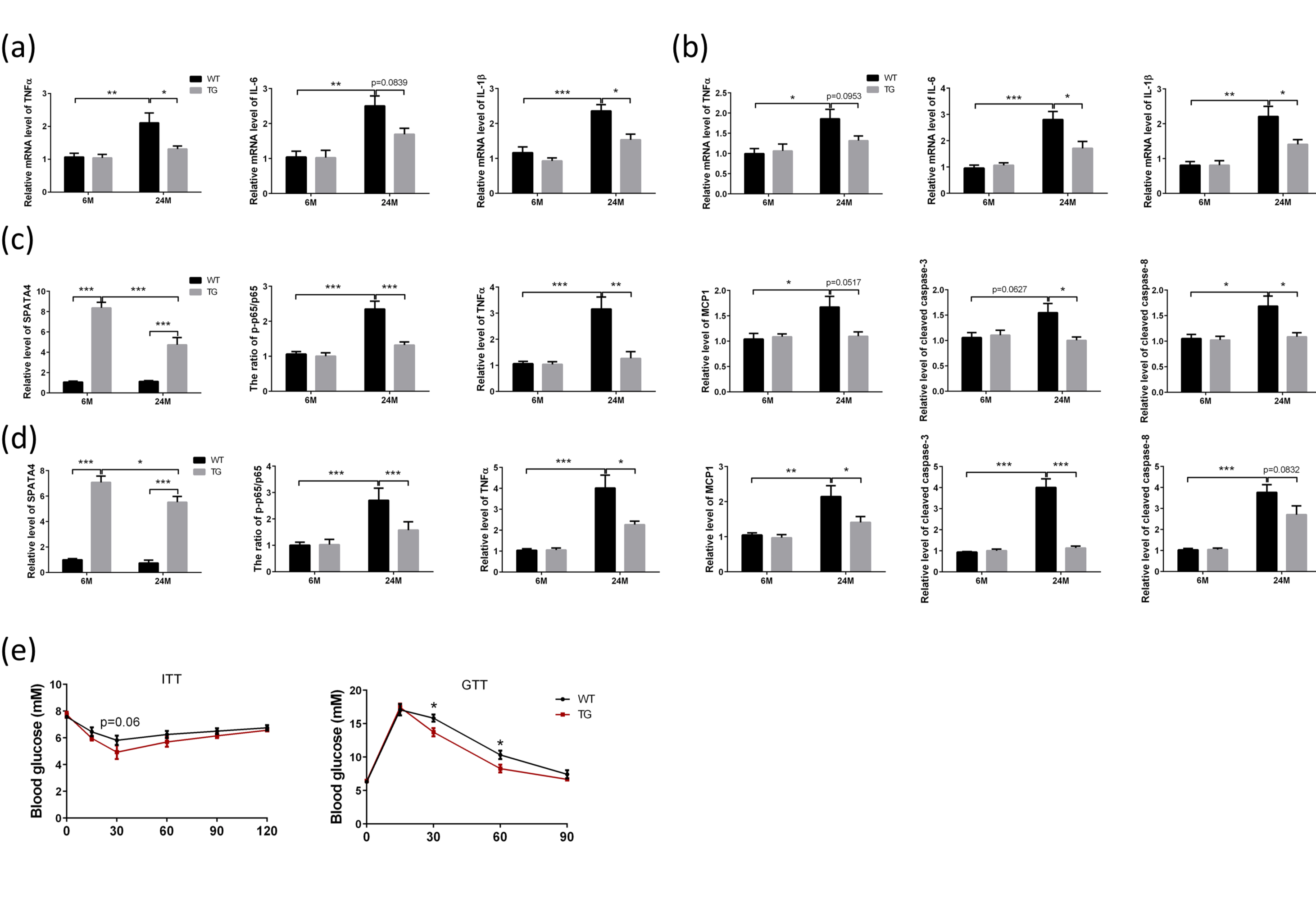

Supplement: Supplementary file 3 — Fig S3 [file ACEL-20-e13282-s003.tif]

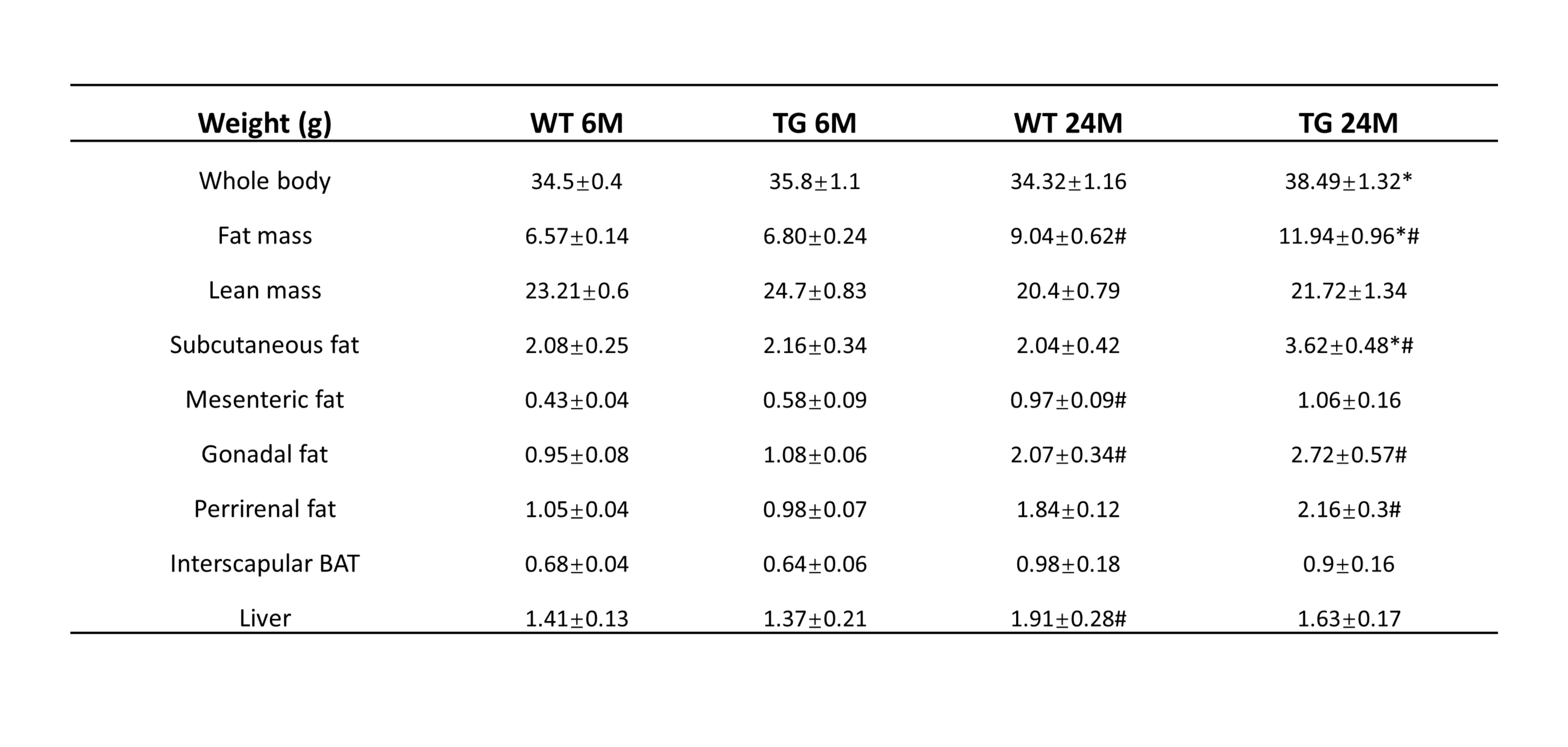

Supplement: Supplementary file 4 — Table S1 [file ACEL-20-e13282-s004.tif]

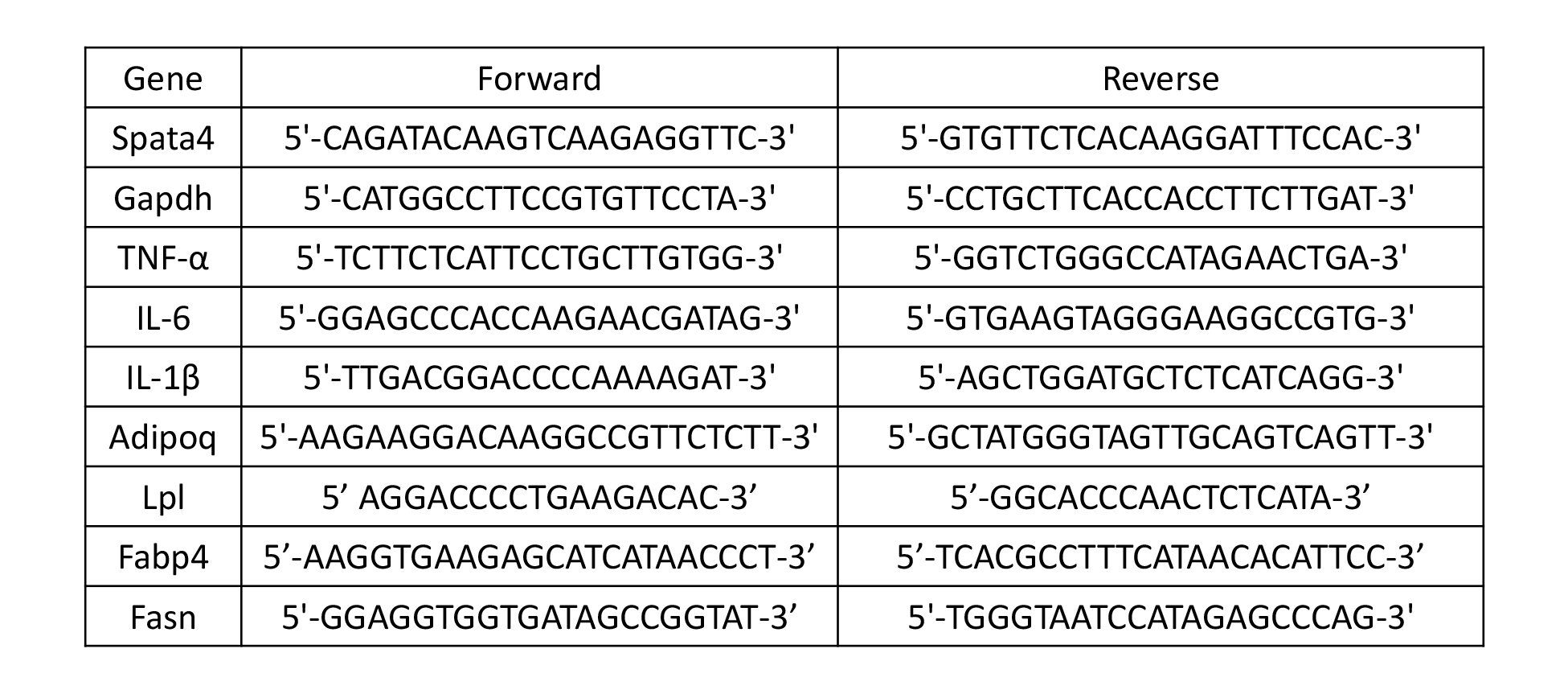

Supplement: Supplementary file 5 — Table S2 [file ACEL-20-e13282-s005.tif]
